# Speed-Accuracy Tradeoff Operator Characteristics of Endogenous and Exogenous Covert Orienting of Attention

**DOI:** 10.1100/tsw.2005.20

**Published:** 2005-02-16

**Authors:** Peter A. McCormick, Lori Francis

**Affiliations:** ^1^ St. Francis Xavier University, Antigonish, NS, Canada; ^2^ St. Mary's University, Halifax, NS, Canada

**Keywords:** orienting attention, endogenous and exogenous orienting, speed-accuracy tradeoff, signal detection theory

## Abstract

There is debate over the mechanisms that govern the orienting of attention. Some argue that the enhanced performance observed at a cued location is the result of increased perceptual sensitivity or preferential access to decision-making processes. It has also been suggested that these effects may be the result of trades in speed for accuracy on the part of the observers. In the present study, observers performed either an exogenous or an endogenous orienting of attention task under both normal instructions (respond as quickly and as accurately as possible) and speeded instructions that used a deadline procedure to limit the amount of time observers had to complete a choice reaction time (CRT) task. An examination of the speed-accuracy operating characteristics (SAOCs) yielded evidence against the notion that CRT precuing effects are due primarily to a tradeoff of accuracy for speed.

## INTRODUCTION

The processes that govern visual attention have been studied extensively. A major area of interest has been the mechanisms involved in the orienting of attention. Posner[1] defined "orienting" as the "aligning of attention with a source of sensory input or an internal semantic structure stored in memory" (p. 4). Several studies have confirmed that we can orient attention toward a stimulus without an accompanying eye movement (e.g., [2]). It is now well established that performance on a variety of detection and discrimination tasks improves when advanced information concerning the probable location of the imperative stimulus is given to an observer.

## The Spotlight Metaphor

There have been several metaphors proposed as explanatory tools to guide research on the orienting of attention. The most widely used is the spotlight metaphor popularized by Posner et al.[2]. This metaphor compares attention to a spotlight that illuminates, in a perceptual sense, the area that its beam encompasses. It provides a *perceptual sensitivity* explanation for the improved performance at attended locations of space. Eriksen and colleagues[3,4] suggested that the spotlight is a "zoom lens" that has variable dimensions that allow for the adjustment of the spatial region that attention can cover[5]. Most proponents of the spotlight model subscribe to the view of a unified focus of visual attention. That is, that attention cannot be allocated to nonadjacent regions. While many experiments that have tested this proposal directly have confirmed it (e.g., [2,6,7]), other theorists (e.g., [8,9,10]) have proposed that attention can be allocated flexibly according the relative probability of the target appearing at different locations. The empirical evidence for this latter position, however, has not been strong[7], and many theorists still hold to the view of a unified focus of attention.

Many of the studies that have investigated the spotlight model have used an experimental design known as the precuing procedure. The use of this procedure is largely the result of an experimental strategy employed by Posner and colleagues[1,2,11]. In a typical precuing experiment, observers are informed as to the most likely location at which a target will occur. Cues that correctly predict the location of the target are called "valid" cues, while cues that incorrectly predict the location of the target are called "invalid" cues. Improved performance as a result of valid cues is referred to as a *benefit*. Inhibition that results from invalid cues is referred to as a *cost*.

## Endogenous and Exogenous Control

The roles of stimulus and cognitive control over the orienting of attention have been studied extensively by making use of an existing dichotomy between two cuing procedures[13,14,15,16,17,18,19,20]. Attention can be captured by a stimulus that appears in the periphery of the visual field. This type of cuing is often referred to as *peripheral* or *exogenous* cuing. Orienting of attention in response to an exogenous cue is involuntary, that is, it occurs without the intention of the observer. Orienting attention in this manner thus appears to be automatic and is suggestive of stimulus control[14,16,21]. A shift of attention can also be the result of a cue that appears at a nontarget location. An example of such a cue would be a central arrow pointing toward the most likely target location. The cue is a symbolic one, therefore, the observer must intentionally interpret the cue and shift his or her attention accordingly. A *central* cue of this type is also known as an *endogenous* cue. Orienting in this manner places the movement of attention under cognitive, rather than stimulus, control. The two mechanisms differ in time course[18,20], with orienting in response to exogenous cues occurring more quickly than orienting in response to endogenous cues. In simple detection tasks, the benefits of exogenous cuing are replaced with costs if the target occurs more than 200 ms after the cue[22], but this "inhibition of return" effect is typically not found with endogenous cues.

Klein and colleagues[23,24,25] suggested that the differences between exogenous and endogenous cuing go beyond the governing mechanisms discussed above. They claimed that the attentional processes that accompany exogenous and endogenous cuing are qualitatively different: exogenous orienting of attention involved the allocation of general visual processing resources to a location, while endogenous orienting involved giving the decision system access to a specific pathway. The notion of decision processes playing a role in the endogenous orienting of attention leads to the suggestion that response bias and not perceptual sensitivity may account for a large proportion of the costs and benefits that are observed in an orienting of attention experiment. A criterion account proposes that observers shift their criterion for the amount of evidence that is needed to indicate detection for cued vs. uncued locations. A valid cue to a particular region of space would decrease the observer's criterion for responding to a target at that location because of the high probability that it will occur there. This shift in criterion may or may not be accompanied by an increase in perceptual sensitivity.

## Perceptual Sensitivity vs. Response Bias

Posner et al.[2] argued against a response bias view of orienting of attention. They claimed that knowing the identity of a target in advance did not facilitate a response, while knowing the location of a target in advance did. This finding is consistent with the notion that sensitivity was enhanced at the cued location. They also noted that the speed-accuracy tradeoff predicted by a response bias view did not occur in choice reaction time (CRT) tasks. In addition, they found that performance at the second most likely location was enhanced not by the likelihood that the target would occur there, but by its spatial proximity to the most likely target location.

The two explanations for improved performance at cued locations, the perceptual sensitivity and the response bias explanations, led researchers to use signal detection theory in an attempt to resolve this issue. Signal detection theory was developed to assess independently changes in sensitivity and response bias[26]. Bashinski and Bacharach[27] argued that the procedure could elucidate the separation of sensory changes and decision changes in orienting of attention tasks. They reasoned that if shifts of attention involved changes in the senses, then they ought to be characterized by changes in the signal plus noise distributions obtained in the experiment (as measured by $d'$). They likewise reasoned that if shifts of attention involved changes in the decision processes, then they ought to be characterized by changes in response bias (as measured by $\beta$). In a study that combined an orienting of attention task with a signal detection analysis, Bashinski and Bacharach found that for detection alone, a valid cue produced sensitivity benefits, while an invalid cue did not produce significant sensitivity costs. When the experimenters employed detection plus localization of the target as a measure of performance, they found that valid cues produced sensitivity benefits and invalid cues produced significant costs. These results supported a perceptual sensitivity explanation for orienting of attention effects.

Müller and Findlay[28] were critical of the analytical methods used by Bashinski and Bacharach[27]. They argued that the manner in which false alarms were assigned for the detection plus localization condition could have produced a sensitivity advantage for cued over uncued locations, even if there was no effect in the data. Archibald[29] noted, however, that even using the alternate method of analysis suggested by Müller and Findlay, the Bashinski and Bacharach sensitivity benefits for cued locations and sensitivity costs for uncued locations holds. In addition, the results of the Müller and Findlay study also agreed with the results of the original Bashinski and Bacharach study. Müller and Findlay concluded that there are sensitivity gains for cued locations (and possibly slight sensitivity losses for uncued locations), but only when the task involved form discrimination. They observed no sensitivity gains for tasks that involved only simple detection. They concluded that manipulating stimulus probability resulted in only a shift of $\beta$ if the task involved detection (i.e., an simple reaction time or SRT task) and a shift in both $d'$ and $\beta$ if the task involved identification (i.e., a CRT task). Downing[30] later demonstrated a sensitivity advantage for simple detection tasks, an effect that was replicated by Müller and Humphreys[31]. Unlike Downing, who argued that the advantage was due to early perceptual quality enhancement, Müller and Humphreys pointed out that the sensitivity advantage might instead be due to a late selection process. Luck et al.[32], however, provided strong evidence against a late selection view of endogenous cuing effects. They combined signal detection psychophysics with electrophysiological measures and found that, for endogenous cues, sensory-related neural responses in visual cortex began as early as 60–100 ms following the presentation of the cue.

## Speed-Accuracy Tradeoff

Identification, which involves CRT, is employed frequently as an alternative to SRT. The procedure allows for error measures that can address valid and invalid trials independently. Furthermore, comparisons between patterns of errors and CRT allow the researcher to detect speed-accuracy tradeoffs that might arise if observers shift their criterion for responding rather than gaining an advantage from improved perceptual sensitivity or preferential access to decision processes. Shiu and Pashler[33] discussed some concerns about the possibility of undetected speed-accuracy tradeoffs in CRT tasks. They pointed out that speed-accuracy tradeoffs might occur and go undetected because observers usually respond at a very high level of accuracy. At this performance level, relatively small decreases in accuracy can be accompanied by large decreases in reaction time. The speed-accuracy operating characteristic (SAOC), presented in Fig. 1, plots reaction time against accuracy of performance. As can be seen in this figure, the level of an observer's performance under normal instructions results in an error rate that is not significantly different from the ideal. The difference in reaction time (RT) that accompanies this decline in performance is, however, significant. Thus, while the statistical sensitivity of an experiment might be sufficient for detecting differences in RT, the differences in errors might be so slight that there is insufficient power to detect them.

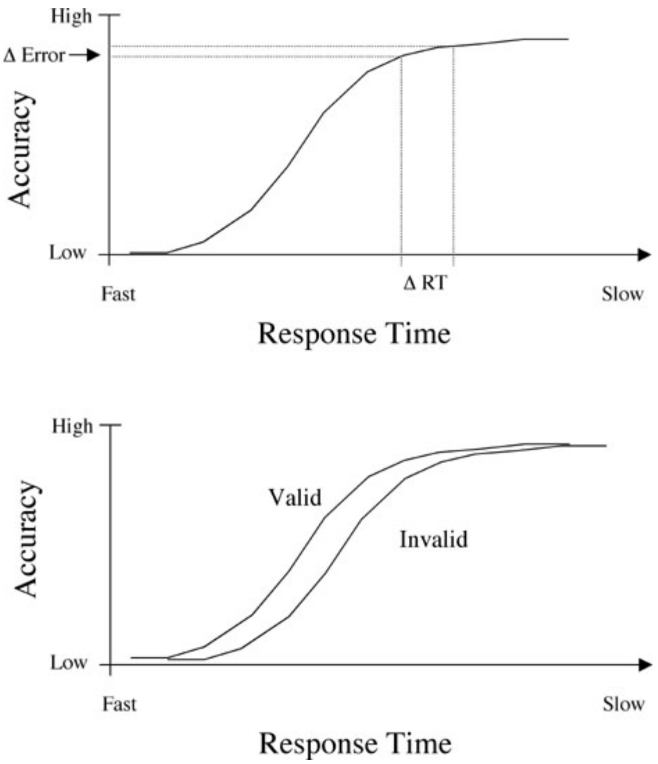

**FIGURE 1.** (Above) The SAOC[38]. At an accuracy level obtained through normal instruction, a small change in the error rate (Δ error) can result in a large change in response time (Δ RT). (Below) The enhanced perceptibility hypothesis predicts two different SAOCs: one for "valid" and one for "invalid" trials.

The instructions given by experimenters in an experimental setting are often antagonistic[34]. Experimenters usually ask observers to maximize both speed and accuracy. Howell and Kreidler[35] noted that this confound can lead observers to assign a criterion to the opposite goals of speed and accuracy. The major concern is that, to the extent that observers are similar in the weights they assign to speed and accuracy, the criterion shifts may be confounded with experimental conditions. Shouten and Bekker[36] found that, as a function of RT, the fraction of errors in performance increased as RT decreased. Moreover, the function relating error rate to RT was identical across different observers.

Researchers tend to think that observers do not rush their responses in an experimental situation[37]. In reality, however, this is often not the case. Observers tend to choose a criterion that rushes their response, but still produces data that are within the acceptable error rate for the experiment. Howell and Kreidler[35] found that observers tended to set a level of performance such that errors did not exceed 4%, which underscores the concern that the tradeoff between speed and accuracy is correlated with the experimental results.

Swensson[38] discussed some of the models put forth to explain the processes that govern the speed-accuracy tradeoff. One group of models is the incremental models. For these models, tradeoffs reflect a partial processing of the stimulus. The tradeoff is contingent on how much stimulus information the observer processes before eliciting a response. The second group of models are the fast guess models[39]. These models propose that an observer has two choices: he or she can make a "fast guess" once the stimulus appears, such a choice results in a high error rate, or the observer can choose to process the stimulus information and thus make a slower and more accurate response. Swensson and Edwards[40] found that these models make different predictions concerning payoff matrices. Swensson suggested an extension of the fast guess model, the deadline model. This model assumes that an observer can choose the fast guess route or the observer can attempt to discriminate the stimulus. If the observer chooses to discriminate, an internal timer allows a certain amount of time for processing. The response is made once the observer has discriminated the stimulus or the internal time limit has run out, whichever situation occurs first. The shorter the deadline, the higher the error rate.

The approach usually taken to explore speed-accuracy tradeoffs is to induce observers to work at various rates of response[37]. Some experimenters have given observers verbal instructions to work at different rates in various blocks of trials[35]. Other experimenters have offered monetary incentives to observers who favored speed for accuracy[38,41]. The procedure that has been found to be most efficient methodologically is the deadline method. An RT limit is defined for the observers and they are encouraged to respond within this time limit. After each trial, observers are given feedback as to whether or not they have beaten the deadline. The deadline is manipulated from block to block in order to produce speed-accuracy shifts[37].

## The Present Study

Wicklegren[42] has contrasted the importance and usefulness of the SOAC with the overwhelming neglect that the method has received. He stated:

> "...it is fair to say that the area of speed-accuracy tradeoff (SAT) is still a special field of its own, the province of a few investigators, with little appreciation that the speed-accuracy tradeoff experiment and the SAT function have great potential to advance all areas of cognitive psychology.[T]he speed-accuracy tradeoff method is so superior to the traditional reaction time method, that many psychologists interested in studying the dynamics of information processing in perceptual, memory, performance, psycholinguistics and other cognitive tasks, ought, in many instances, to do speed-accuracy tradeoff studies instead of reaction time studies... The case for speed-accuracy tradeoff... against reaction time is so strong that this case needs to be presented as forcefully as possible to all cognitive psychologists." (p.68)

Despite the strong recommendation of Wickelgren[42] and the nature of the controversies presented earlier, which seem to call for a speed-accuracy tradeoff analysis of cuing effects, few have yet been done. Carrasco and colleagues are a rare exception, although they have focused primarily on search tasks[43,44]. The present study was designed primarily to establish whether cuing effects in CRT tasks are due to speed-accuracy tradeoff or to a genuine advantage in processing efficiency. The deadline method was used to study the effects of speed-accuracy tradeoff in exogenous orienting of attention in

Experiment 1 and endogenous orienting of attention in Experiment 2. Observers identified a visually presented stimulus as an “X” or an “O”. The deadlines were enforced by means of a message at the beginning of each block of trials. The message informed observers of the amount of the time they had to respond in each trial. If the observers did not meet this deadline, a message asking them to go faster appeared after their response. It was hypothesized that as the time observers had to respond decreased, so would the accuracy of their performance. It was anticipated that this experiment would shed light on the questions raised by Shui and Pashler[33] concerning alternative explanations of orienting attention effects in RT tasks. If facilitation of performance in response to a precue is due to an increase in perceptual sensitivity or preferential access to decision-making processes, then two different SAOCs ought to exist for the two types of cue-target relationships (i.e., valid and invalid cues, see Fig. 1). More specifically, the SAOC for valid cues ought to be faster than the SAOC for invalid cues. Such a shift along the reaction time axis is suggestive of faster processing for valid trials. If cuing effects are due solely to a criterion shift that results in a trade of speed for accuracy, then there ought to be only one SAOC that describes performance on both valid and invalid trials.

## EXPERIMENT 1

Experiment 1 examined the SAOCs for exogenous cuing. The experiment was similar to many exogenous orienting of attention experiments. Observers were given no advance information concerning where the potential target (an “X” or an “O”) would occur. One of two boxes marking the two possible target locations brightened briefly just prior to the arrival of the target. Observers participated in four sessions. Each session consisted of several deadline blocks of trials. In the first block, observers were given unlimited time to respond, but were informed that they ought to respond as quickly and as accurately as possible (normal instructions). Following this block, observers were given decreasing deadline intervals, ranging from 590–200 ms within which they were to make their response. If exogenous cuing effects are due solely to a speed-accuracy tradeoff, there ought to be no difference in the SAOCs for valid and invalid cues. If, on the other hand, exogenous cuing effects are due to some other mechanism that results in faster processing (i.e., enhanced perceptual sensitivity or preferential access to decision-making processes), then the SAOC for valid trials ought to be shifted down the RT axis from the operating characteristic for invalid trials.

### Method

#### *Observers*

Eight students from St. Francis Xavier University participated in this experiment. The observers received class credit for their participation and were paid $15 at the end of the study as an extra incentive to complete all four sessions. All observers had normal or corrected to normal vision.

#### *Stimuli and Materials*

The background of the computer remained black for the duration of the experiment. The fixation point, which appeared in the center of the screen, was a plus sign ($0.4° \times 0.4°$ visual angle). One outlined box ($2° \times 2°$ visual angle) appeared 5° visual angle to the right and another 5° to the left of the fixation point. The exogenous cue was a flash of one of these boxes that lasted for 30 ms. The target occured 60 ms later and was either an “X” or an “O” (0.7° visual angle in height $\times$ 0.6° visual angle in width) that appeared in one of the two boxes. The stimuli were presented on a 14-in. AppleColor High-Resolution RGB monitor, controlled by a Macintosh Quadra 700 microcomputer.

### Procedure

Observers took part in four experimental sessions. In each session, observers sat facing the monitor with their heads supported by a chin rest located 57 cm from the front of the computer screen. At the beginning of the first session, observers were fully informed as to the purpose and procedure of the experiment. At the beginning of each session, observers completed a block of 50 trials where they were asked to simply identify "Xs" or "Os" as they appeared on the screen. They were informed that the brightening of a box was unrelated to the location of the impending target. They were instructed that they ought to perform as quickly and as accurately as possible. The cutoff for this block was 5,000 ms and served as a "normal instructions" condition. Observers responded by depressing one of two preselected keys on the keyboard. After the 50 normal instructions trials, observers began the speeded trials. They were told that if they used more than the allotted time, a message would appear on the screen, indicating that the next response ought to be faster. As in the first block of trials, observers were also informed that the box flashes were distractors that would not offer any clues concerning the location of the impending target. The observers were asked to fix their gaze on the fixation stimulus while dispersing their attention across both boxes.

Each session consisted of 14 blocks of 50 trials each. The cutoff rate for responding was 590 ms in the first block and decreased by decrements of 30 ms in each of the remaining blocks. Thus, the cutoff rate in the fourteenth and final block was 200 ms. Each block consisted of 50% valid trials and 50% invalid trials. Of the targets, 50% were "Xs" and 50% were "Os". Each trial began with the appearance of the fixation stimulus, which remained on the screen for the duration of the trial. The peripheral cue then flashed for an interval of 30 ms. After an interstimulus interval of 67 ms, the target appeared as an "X" or an "O" in one of the boxes. They identified the target as an "X" or an "O" by depressing a response key on the computer keyboard. Four of the observers responded "X" with their dominant hand, while four responded "O" with their dominant hand.

## Results

### Normal Instructions

The RT and error data were analyzed in two separate repeated measures analysis of variance (ANOVA) that used cue validity (valid or invalid) as the independent variable. Under normal instructions, observers responded more quickly following valid cues (698 ms) than invalid cues (742 ms), $F(1, 7) = 15.62$, $MSe = 475.99$, $p < 0.006$. There was no significant difference between the error rates for valid trials (2.4%) and invalid trials (1.8%).

### Deadline Instructions

#### Response Times

The RT data presented in Appendix A were subjected to a repeated measures ANOVA that included the factors of cue type (valid-invalid) and cutoff (14 deadlines). There was a main effect of cutoff, $F(13, 91) = 91.98$, $MSe = 1378.90$, $p < 0.001$. There was also a main effect of cue type, with observers responding faster following valid cues (322 ms) than following invalid cues (328 ms), $F(1, 7) = 50.32$, $MSe = 39.76$, $p < 0.001$. There was a significant interaction between the two variables, $F(13, 91) = 2.32$, $MSe = 97.82$, $p < 0.01$. Observers tended to perform consistently faster on valid trials in the earlier, slower intervals, but there was little or no difference between valid and invalid trials in the fastest intervals.

## Error Rates

The error data presented in Appendix A were subjected to a repeated measures ANOVA that included the factors of cue type (valid-invalid) and cutoff (14 deadlines). There was a main effect of cutoff, $F(13, 91) = 176.81$, $MSe = 23.95$, $p < 0.001$, but there was no significant main effect of cue type, and no significant interaction, $p > 0.23$.

### *Interval Analysis*

## ANOVA

The valid cue and invalid cue RT data for both correct and incorrect responses were partitioned into 14 intervals each. Each observer's data were ranked from the fastest to the slowest responses within each of the two cue conditions. Intervals containing 100 responses were selected from the ranked data for each of the two cue conditions. The mean error rates and response times were then calculated for intervals within each of the cue conditions. These data are presented in Table 1. The RT and error data were each subjected to a repeated measures ANOVA that included the factors of cue type (valid-invalid) and interval (14 intervals). Because of the manner in which the data were partitioned, there was an expected main effect of interval for RT, which was significant, $F(13, 91) = 340.3$, $MSe = 773.49$, $p < 0.001$. More importantly, there was also a main effect of cue, $F(1, 7) = 74.7$, $MSe = 44.65$, $p < 0.001$, with faster interval means for valid trials than for invalid trials (317.5 vs. 325.3 ms, for valid and invalid cues, respectively). There was also an interaction between interval and cue, $F(13, 91) = 4.54$, $MSe = 40.87$, $p < 0.001$. As evidenced in Table 1, this interaction likely comes about because the difference between cues becomes more pronounced as the intervals become comprised of slower responses.

**TABLE 1**
**Mean Response Times (ms) and Error Rates (in Brackets) Across Equal-Sized Intervals of the Ranked RT Data in Experiment 1 (Each Cell Contains 100 Trials)**

| | Cue Condition | | |
|---|---|---|---|
| Cut-off | Valid | Invalid | Difference |
| 14 | 549 (.025) | 575 (.028) | 26 (.003) |
| 13 | 452 (.031) | 462 (.043) | 10 (.012) |
| 12 | 419 (.046) | 430 (.055) | 11 (.009) |
| 11 | 396 (.070) | 408 (.071) | 12 (.001) |
| 10 | 377 (.084) | 389 (.089) | 12 (.005) |
| 9 | 360 (.106) | 370 (.106) | 10 (.000) |
| 8 | 341 (.125) | 350 (.131) | 9 (.006) |
| 7 | 321 (.168) | 327 (.186) | 6 (.018) |
| 6 | 296 (.241) | 301 (.268) | 5 (.027) |
| 5 | 268 (.320) | 272 (.303) | 4 (-.017) |
| 4 | 238 (.370) | 241 (.380) | 3 (.010) |
| 3 | 202 (.451) | 204 (.451) | 2 (.000) |
| 2 | 150 (.468) | 151 (.455) | 1 (-.013) |
| 1 | 76 (.465) | 73 (.486) | -3 (.021) |

For error rates, there was also a significant effect of interval, $F(13, 91) = 84.0$, $MSe = 54.75$, $p < 0.001$. There was no significant difference in error rates between cue conditions (21.2 vs. 21.8%, for valid and invalid cues, respectively), $F(1, 7) = 1.58$, $MSe = 11.25$, $p > 0.24$, nor did cue interact with interval ($F < 1.0$).

### Speed-Accuracy Operating Characteristics

SAOCs were determined by plotting the mean RT for intervals against the odds of making a correct response (i.e., the probability of a correct response divided by the probability of making an incorrect response; see Pew, 1969 [45]). The SAOCs for valid and invalid trials are presented in Fig. 2. The data were trimmed to more closely examine the effect of cuing over a portion of the operating characteristic where a tradeoff was occurring. From Fig. 2, it can be seen that the two fastest intervals and the slowest interval deviate substantially from the linearity of the remaining data points. These three points were removed from each of the two cue conditions and the best fitting lines were determined by regressing the logarithm of the odds of making a correct response on the mean of the ranked RT interval data. As can be seen in Fig. 2, the trimmed data were well fit by the regression lines ($r^2 = 0.992$ for valid trials, $r^2 = 0.990$ for invalid trials). It can also be seen that the valid cue SAOC was steeper than the invalid SAOC. This interaction is analogous to the interaction between interval and cue condition obtained in the RT portion of the previous ANOVA. To ensure that the interaction between cue and interval also occurred within the trimmed data, one ANOVA was performed on the trimmed portion of the interval mean RTs and another ANOVA was performed on the interval error rates. Referring to Table 1, this analysis included 11 levels of intervals ranging from Interval 3 through Interval 13 and cue condition (valid or invalid) as variables. The results paralleled the results of the previous ANOVAs. The mean RT for valid trial intervals was faster than for invalid trials (334 vs. 341 ms, $F[1, 7] = 42.7$, $MSe = 60.23$, $p < 0.001$). Cue condition also interacted with interval, $F(10, 70) = 7.93$, $MSe = 7.60$, $p < 0.001$. To explore this interaction further, a polynomial contrast was performed comparing the slope of the linear component of interval for valid trials with the slope of linear component of interval for invalid trials. The slopes differed significantly, $F(1, 7) = 12.14$, $MSe = 42.25$, $p < 0.02$. The mean RT for invalid intervals increased about 1 ms per interval more than the mean RT for valid trial intervals. For error rates, only a main effect of interval was observed, $F(10, 70) = 67.7$, $MSe = 56.13$, $p < 0.001$. All other effects and interactions, including a polynomial contrast across cue conditions, were nonsignificant (all $F < 1.0$).

The difference scores, listed in Table 1, represent the size of the cuing effect across different intervals. Because they are difference scores, they also represent the interaction between cuing and interval. These data, plotted in Fig. 3, were fit using a linear regression. As expected by the results of the polynomial contrasts, the RT difference data were fit well, $r^2 = 0.853$, and the error difference data were fit very poorly, $r^2 = 0.016$. The regression lines are presented in Fig. 3. The size of the RT cuing effect gradually gets smaller as the RT intervals become comprised of faster responses, while errors are distributed randomly around a (nonsignificant) cue size effect of 0.6%.

## Discussion

There are three important findings in the present experiment. First and foremost, exogenous cuing effects in CRT tasks cannot be explained solely by a tradeoff in accuracy for speed as evidenced by the two distinct SAOCs observed for valid and for invalid trials. A sizable portion of the exogenous cuing effect, however, may be due to small tradeoffs when normal speed and accuracy instructions are used. Finally, the exogenous cuing effect appears to be primarily an RT effect with a lack of any significant effects involving cuing in all of the above analyses of error rates.

If exogenous cuing effects in CRT tasks were due solely to a speed-accuracy tradeoff, then we would expect valid and invalid trials to follow the same SAOC. The present experiment, however, yielded different processing efficiencies as reflected in the different SAOCs for the two cue conditions. This finding need not imply that no tradeoff can occur under conditions of normal instructions. In fact, the results of the present experiment suggest that tradeoffs under normal instructions can have a sizable effect on the RT difference. A 44-ms priming effect was observed in the normal instructions condition. This RT advantage was accompanied by a nonsignificant difference in errors (2.4% for valid vs. 1.8% for invalid). The size of the RT cuing effect is larger than expected from the size of the difference in the

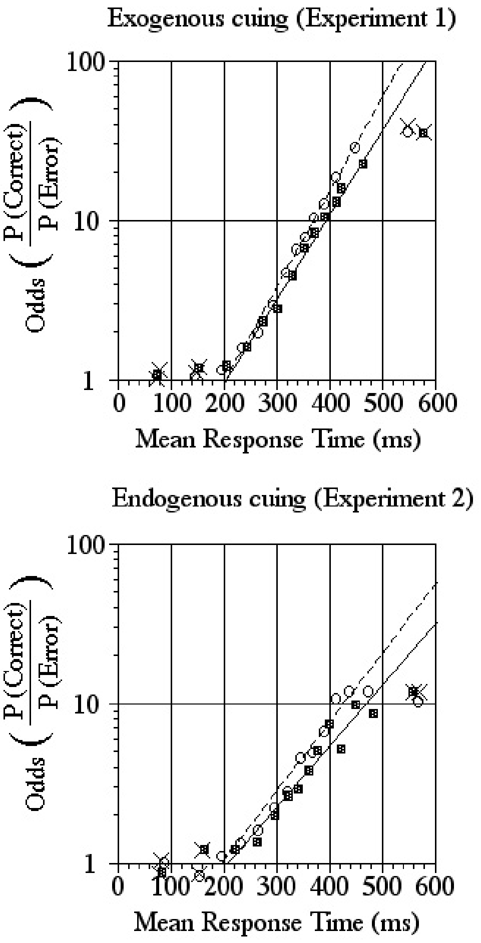

**FIGURE 2.** SAOCs for valid and invalid cues in Experiment 1 (exogenous cues) and Experiment 2 (endogenous cues). The data, trimmed across a range where a tradeoff occurred (symbols without a superimposed "X"), were fit by regressing the logarithm of the odds of making a correct response on the mean of the ranked RT interval data. Dashed lines and open circles represent valid cues, solid lines and closed squares represents invalid cues.

SAOCs. Even at the widest separation across the SAOCs, there is less than a 25-ms difference (see Fig. 2). To calculate the size of the potential contamination of the RT data from this small tradeoff, the regression equations presented in Fig. 2 were used to estimate the expected difference in RT. For the SAOC associated with valid trials, the shift from 2.4 to 1.8% is expected to result in a 21-ms change in RT; for the SAOC associated with invalid trials, the shift in error rates translates into a 24-ms shift. Thus, as much as half of the RT cuing effect observed under conditions of normal instructions may have been due to a tradeoff for a very small amount of accuracy. Research on exogenous orienting of attention that finds significant RT differences, but not significant error rate differences, may be reporting more than an exogenous cuing effect. These findings are consistent with the warning put forth by Shiu and Pashler[33] that speed-accuracy tradeoffs may go undetected in the exogenous orienting task. Caution ought to be taken when considering the instructions given to observers in CRT tasks because observers can set varying criteria for speed and accuracy[36]. In the present experiment, we were able to determine that the two types of cues, while leading to different criteria, were also associated with different SAOCs.

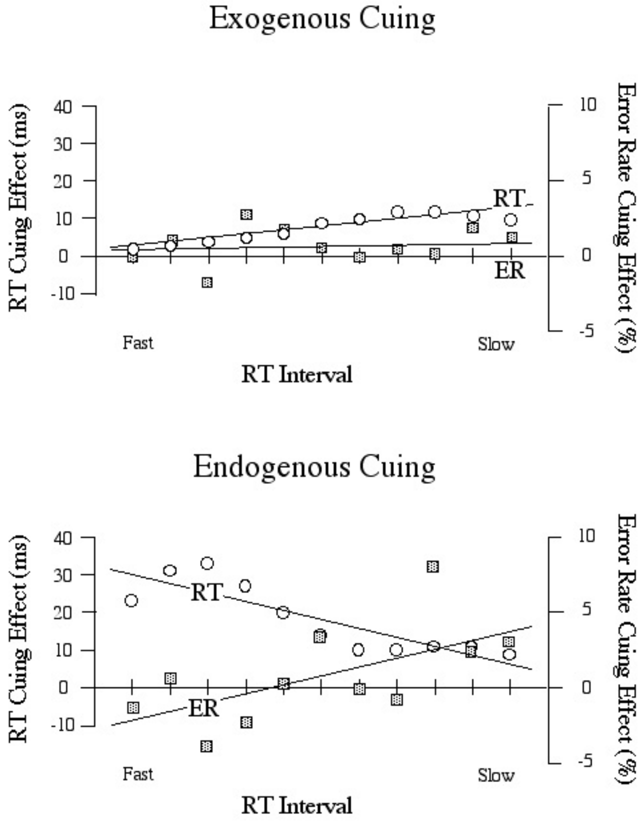

**FIGURE 3**. Cuing effects (invalid-valid) in Experiment 1 (exogenous cues) and Experiment 2 (endogenous cues) across RT intervals. Open circles represent RTs, shaded squares represent errors.

A potentially important result in the present experiment is that all of the significant cuing effects were found in analyses of RT. The analysis of error rates yielded no cuing effects or interactions. It is tempting to claim that the advantage RT has in uncovering cuing effects is related to the problem several researchers have encountered in finding cuing effects using accuracy and masked displays. The relationship between RT and error, however, is likely more complicated than this simple explanation. First, there is the present finding that the size of the (RT) cuing effect is related to the overall level of performance as measured by accuracy. More specifically, the higher the accuracy, the larger the size of the cuing effect. This result may be due to a number of factors. A reasonable explanation is that on some trials, observers decide how they will respond before the target actually appears and make an SRT response based on this decision. If we assume that the number of trials in which this strategy occurs is related to the overall performance level (or alternatively to the amount of time that observers have to respond), then the increasing size of the cuing effect with increasing levels of accuracy is likely due to fewer and fewer of these “fast guess” trials getting mixed in with “true” CRT trials. When observers are responding fastest, the majority of the trials are fast guess trials, effectively eliminating any advantage offered by the cue. When observers are responding slowly and accurately, cuing effects afford an (RT) advantage for targets occurring at the cued location. Several theories on the nature of exogenous cuing effects were discussed earlier, but only two can adequately explain the present pattern of data. As discussed earlier, if exogenous cuing effects are due to enhanced perceptual sensitivity or are due to preferential access to decision-making processes, then an RT advantage must occur for valid trials. Unfortunately, the present experiment cannot distinguish between these two accounts. Noise reduction, on the other hand, does not adequately explain the present results. Because no masking was used, there was no noise to reduce. Thus, while noise reduction can account for the pattern of results that have been observed in masking studies, the theory falls short of explaining the pattern of results found in CRT tasks.

## EXPERIMENT 2

Experiment 2 examined the SAOCs for endogenously oriented attention. As discussed earlier, several researchers have suggested that endogenous and exogenous orienting may involve different mechanisms (e.g., [23,24,25]). There is an intuitive reason for believing that endogenous orienting might be more susceptible to bias effects. In these experiments, the probability of a target occurring at a particular location is manipulated by the experimenter. This method helps to ensure that observers orient their attention to cued locations. Note that in the previous experiment, there was no need for such a probability manipulation because exogenous orienting is reflexive and direct cognitive control is not necessary for its operation. Thus, in the previous experiment, there was no reason for subjects to trade speed for accuracy when a target appeared at an expected location (i.e., following a valid cue) because there was no expected location. The target was equally likely to appear at either location. The design of Experiment 2 was almost identical to the design of Experiment 1. Boxes again marked potential target locations. The cue, however, was an arrow pointing to one of the boxes. The target then appeared at the cued location 75% of the time. Because endogenous and exogenous orienting are thought to follow different time courses, observers were given 500 ms to orient their attention rather than the 90 ms used in Experiment 1. Because of this additional time and the potential for the confounding effects of overt shifts of attention, it was necessary to monitor eye movements. The predictions of Experiment 2 are the same as Experiment 1. If endogenous orienting effects in CRT tasks are due to a speed-accuracy tradeoff, then we expect the same SAOC for valid and invalid trials. If, on the other hand, endogenous orienting effects are due to either enhanced perceptual processing or preferential access to decision-making processes, then we expect valid and invalid cues to operate under different SAOCs.

### Method

#### *Observers*

Eight students from St. Francis Xavier University participated in this experiment. The observers received payment of $50 for their participation. All observers had normal or corrected to normal vision.

#### *Stimuli and Materials*

The stimuli and materials were identical to Experiment 1 with the following exceptions. First, the cue was an arrow that replaced the fixation stimulus. The arrow pointed either to the left or to the right box and informed the observers as to the most likely target location. Eye position was monitored using an ISCAN RK-416 video-based eye-tracking system interfaced with the Macintosh Quadra 700 computer through a National Instruments NB-DIO 24 data collection card. Eye movements in excess of 1º visual angle were detected and discarded without replacement.

#### *Procedure*

The procedure was identical to Experiment 1 with the following exception. Each block consisted of 75% (36) valid trials and 25% (12) invalid trials. In total, observers participated in 2,880 trials. Each trial began with the appearance of the fixation stimulus, which was replaced by the arrow cue after 1 s. The central cue remained on the screen for the duration of the trial. After an interstimulus interval of 500 ms, the target appeared.

## Results

### *Eye Errors*

Eye errors were recorded on 14% of all trials. While this appears quite high, there are several reasons why this many errors were detected. The sensitivity of the equipment was such that table and body movements were often mistaken by the system for eye errors. Given the time pressure that observers were under in many of the conditions of this experiment, sudden body movements were common. The experimenter, who was present with the observer and monitored a video display of his or her eyes, reported no cases where the system missed an eye movement. A conservative approach was used and although many of the eye errors were not true eye errors (i.e., a saccade towards the cued location), any trial on which an eye error was recorded was discarded.

### *Normal Instructions*

The RT and error data were analyzed in two separate repeated measures ANOVA that used cue validity (valid or invalid) as the independent measures. Under normal instructions, observers responded marginally more quickly following valid cues (447 ms) than invalid cues (464 ms), $F(1, 7) = 3.69$, $MSe = 304.5$, $p < 0.10$. There was no significant difference between the error rates for valid trials (10.5%) and invalid trials (10.9%; $F < 1$).

### *Deadline Instructions*

#### Response Times

The RT data presented in Appendix B were subjected to a repeated measures ANOVA that included the factors of cue type (valid-invalid) and cutoff (14 deadlines). There was a main effect of cutoff, $F(13, 91) = 29.09$, $MSe = 3142.5$, $p < 0.001$. There was also a main effect of cue type, with observers responding faster following valid cues (325 ms) than following invalid cues (344 ms), $F(1, 7) = 19.51$, $MSe = 1013.1$, $p < 0.01$. There was no significant interaction between the two variables, $F(13, 91) = 1.34$, $MSe = 309.9$, $p > 0.20$. As can be seen from the data in Appendices B, observers tended to perform consistently faster on valid trials than on invalid trials.

#### Error Rates

The error data presented in Appendix B were subjected to a repeated measures ANOVA that included the factors of cue type (valid-invalid) and cutoff (14 deadlines). There was a main effect of cutoff, $F(13, 91) = 45.49$, $MSe = 64.13$, $p < 0.001$, but no main effect of cue. On average, observers responded correctly on 27.0% of both valid and invalid trials. It can be seen in Appendices B, however, that observers responded more accurately following valid cues in the longer cutoff intervals and more accurately following invalid cues in the shorter cutoff intervals. This trend was reflected in a significant interaction between cue and cutoff, $F(13, 91) = 1.92$, $MSe = 39.08$, $p < 0.05$.

## Interval Analysis

### ANOVA

The valid cue and invalid cue RTs from both correct and incorrect responses were partitioned into 14 intervals each. Each observer's data were ranked from the fastest to the slowest responses within each of the two cue conditions. Unlike Experiment 1, in which observers received an equal number of valid and invalid trials, Experiment 2 had a cue probability manipulation and an unequal number of valid and invalid trials. In addition, a variable number of trials were lost to eye movements. These factors prevented a straight partition of the data into cells with $n$ = 100. Each observer's ranked data were partitioned into equal sized intervals. On average, valid cues yielded intervals with $n$ = 129 and invalid cues yielded intervals with $n$ = 43. The mean error rates and RTs were then calculated for these intervals. The data are presented in Table 2. The RT and error data were each subjected to a repeated measures ANOVA that included the factors of cue type (valid-invalid) and interval (14 intervals). Because of the manner in which the data were partitioned, there was an expected main effect of interval for RT, which was significant, $F(13, 91) = 230.6$, $MSe = 1157.2$, $p < 0.001$. More importantly, there was also a main effect of cue, $F(1, 7) = 11.12$, $MSe = 975.6$, $p < 0.05$, with faster interval means for valid trials than for invalid trials (320.3 vs. 334.2 ms, for valid and invalid cues, respectively). There was also an interaction between interval and cue, $F(13, 91) = 8.01$, $MSe = 65.27$, $p < 0.001$. As evidenced in Table 2, this interaction likely comes about because the difference between cues becomes more pronounced as the intervals become comprised of slower responses.

For error rates, there was also a significant effect of interval, $F(13, 91) = 84.5$, $MSe = 50.04$, $p < 0.001$. There was no significant difference in error rates between cue conditions (26.1 vs. 26.3%, for valid and invalid cues respectively; $F < 1.0$), but cue did interact with interval, $F(13,91) = 2.123$, $MSe = 29.59$, $p < 0.05$. It can be seen in Appendices B that this interaction likely comes about because of the tendency for observers to have responded more accurately following valid cues in intervals comprised of slower responses and more accurately following invalid cues in the intervals comprised of faster responses.

**TABLE 2**
**Mean Response Times (ms) and Error Rates (in Brackets) Across Equal-Sized Intervals of the Ranked RT Data in Experiment 2 (Each Cell Contains 100 Trials)**

| | Cue Condition | | |
|---|---|---|---|
| Cut-off | Valid | Invalid | Difference |
| 14 | 562 (.078) | 556 (.079) | 6 (.001) |
| 13 | 470 (.075) | 479 (.105) | 9 (.030) |
| 12 | 433 (.076) | 444 (.093) | 11 (.017) |
| 11 | 406 (.084) | 417 (.164) | 11 (.080) |
| 10 | 385 (.128) | 395 (.120) | 10 (-.008) |
| 9 | 364 (.166) | 374 (.165) | 10 (-.001) |
| 8 | 341 (.176) | 355 (.209) | 14 (.033) |
| 7 | 317 (.256) | 337 (.258) | 20 (.002) |
| 6 | 291 (.303) | 318 (.280) | 27 (-.023) |
| 5 | 260 (.377) | 293 (.338) | 33 (-.039) |
| 4 | 227 (.424) | 258 (.430) | 31 (.006) |
| 3 | 193 (.470) | 216 (.456) | 23 (-.014) |
| 2 | 153 (.541) | 160 (.452) | 7 (-.089) |
| 1 | 81 (.493) | 78 (.534) | -3 (.041) |

### Speed-Accuracy Operating Characteristics

SAOCs were determined by plotting the mean RT for intervals against the odds of making a correct response, as was done in Experiment 1. The SAOCs for valid and invalid trials are presented in Fig. 2. The data were trimmed to more closely examine the effect of cuing over a portion of the operating characteristic where a tradeoff was occurring. As in Experiment 1, the two fastest intervals and the slowest interval were removed from each of the two cue conditions and the best fitting lines were determined by regressing the logarithm of the odds of making a correct response on the mean of the ranked RT interval data. As can be seen in Fig. 2, the trimmed data were well fit by the regression lines ($r^2 = 0.969$ for valid trials, $r^2 = 0.935$ for invalid trials). It can also be seen that the valid cue SAOC was steeper than the invalid SAOC. This interaction is analogous to the interaction between interval and cue condition observed in the RT portion and, unlike Experiment 1, in the error portion of the previous ANOVA. To ensure that the interactions between cue and interval also occurred within the trimmed data, one ANOVA was performed on the trimmed portion of the interval mean RTs and another ANOVA was performed on the interval error rates. Referring to Table 2, this analysis included 11 levels of intervals ranging from Interval 3 through Interval 13 and cue condition (valid or invalid) as variables. The mean RT for valid trial intervals was faster than for invalid trials (335 vs. 353 ms, $F[1, 7] = 13.00$, $MSe = 1079.5$, $p < 0.01$). Cue condition also interacted with interval, $F(10, 70) = 7.75$, $MSe = 41.85$, $p < 0.001$. To explore this interaction further, a polynomial contrast was performed comparing the slope of the linear component of interval for valid trials with the slope of linear component of interval for invalid trials. The slopes differed significantly, $F(1, 7) = 10.97$, $MSe = 217.4$, $p < 0.02$. The mean RT for invalid intervals increased about 1 ms per interval more than the mean RT for valid trial intervals. For error rates, only a main effect of interval was observed, $F(10, 70) = 63.58$, $MSe = 46.00$, $p < 0.001$. There was no main effect of cue ($F < 1$) and unlike the untrimmed interval data, there was no interaction between cue and interval, $F(10, 70) = 1.38$, $MSe = 30.41$, $p > 0.20$. A polynomial contrast comparing the linear component of interval across cue conditions, however, did approach significance, $F(1, 7) = 3.94$, $MSe = 39.37$, $p < 0.09$.

The cuing by interval interactions was further explored by performing a linear regression on the difference scores listed in Table 2. These data and the best fitting lines for RTs and errors are presented in Fig. 3. As expected by results of the polynomial contrasts, the RT difference data were fit well, $r^2 = 0.743$, and the error difference data was moderately fit, $r^2 = 0.347$. The size of the RT cuing effect gradually gets larger as the RT intervals become comprised of faster responses, while the cuing effect in errors gradually gets smaller. The effect may even flip over to an invalid cue advantage in the fastest response intervals, although there is no solid statistical evidence that this reversal is occurring (recall that the interaction between cue and interval in the polynomial contrast was only marginally significant).

## Discussion

As in Experiment 1, there are three important findings in the present experiment. First, as was found with exogenous orienting of attention, endogenous cuing effects in CRT tasks cannot be explained solely by a tradeoff in accuracy for speed as evidenced by the two distinct SAOCs observed for valid and for invalid trials (Fig. 2). Unlike exogenous orienting, there was no tradeoff evidenced when normal speed and accuracy instructions are used. There was, however, evidence of a tradeoff occurring in the fastest RT intervals. Finally, the endogenous cuing effect appears to involve both an RT advantage and a flexible use of accuracy that has a substantial effect on the size of that advantage.

There are two lines of evidence supporting the notion that endogenous cuing effects in CRT are not due solely to speed-accuracy tradeoff. First, the SAOCs for valid and invalid trials presented in Fig. 2 are clearly different. Second, across the slower RT intervals, there is a valid cue advantage in RT that is associated with a valid cue advantage in accuracy (see Fig. 3). It does appear, however, that when observers are responding quickly because they are under time pressure, they begin to trade speed for accuracy. Unlike Experiment 1, where we suggested that fast guess trials tended to occur in both valid and invalid trials, in the present experiment, where the target was three times more likely to occur at the cued location than at the uncued location, fast guess trials were probably more likely to occur on valid trials. A simple bias in responding that is undoubtedly due to the observer's expectancy.

## GENERAL DISCUSSION

The present research makes both theoretical and methodological contributions to our understanding of the mechanisms involved in the orienting of attention. At a theoretical level, the data are quite consistent with the results of signal detection studies of orienting attention discussed earlier. The results are also consistent with the view that endogenous and exogenous cuing effects represent different mechanisms. Cuing effects in exogenous orienting are primarily an RT effect, while cuing effects in endogenous orienting are a combination of an RT advantage with flexible, but potentially response biased, accuracy. At a methodological level, it can be seen that tradeoffs in speed and accuracy may occur under conditions of normal instructions (for exogenous orienting) and across task difficulty conditions (for endogenous orienting). Careful consideration ought to be given to the task parameters that define an exogenous or an endogenous orienting of attention experiment, particularly the relationship between errors and RT that occur across cuing conditions or other experimental manipulations.

Shui and Pashler[33] suggested that an RT advantage for valid trials might occur because of either a speed for accuracy tradeoff or to preferential access to decision-making processes afforded cued items. The present research has demonstrated clearly that a tradeoff, on its own, cannot account for the RT cuing effect. This finding leaves preferential access and enhanced perceptual sensitivity as viable explanations. While the present experiments were not designed to distinguish between these two alternatives, we believe that the data are consistent with the model proposed by Klein[23,24,25]. In this view, exogenous orienting effects are thought to be due to enhanced perceptual sensitivity and endogenous cuing effects are thought to be due to preferential access to decision-making processes. It could also be argued that the different SOAC for valid and invalid trials demonstrated for both exogenous and endogenous cues supports the electrophsysiological findings of Luck et al.[32] of sensitivity gains for endogenous orienting. Thus, we would suggest that in addition to enhanced perceptual sensitivity, which occurs for both exogenous and endogenous orienting, there is also an element preferential access that may occur at a later decision stage. This notion is consistent with the dissociation of exogenous and endogenous orienting mechanisms as automatic and controlled processes; i.e., that exogenous orienting is an early, low-level, stimulus-driven process and endogenous orienting is a later, decision- and response-level, cognitively controlled process. The results of Experiment 1 are consistent with this view. There is no evidence of tradeoffs or response biases across RT deadlines or intervals. There is simply a small RT advantage for cued trials that becomes smaller as speed pressure leads to accuracy performance approaching chance levels. This decline is probably due to the increasing number of fast guesses that go with the faster responding.

Experiment 2 yielded evidence that speed and accuracy tradeoffs may play a role in endogenous orienting, particularly under conditions of time pressure. When observers are responding within shorter deadline intervals, and consequently faster, they show a larger RT advantage for cued items, but at a cost in accuracy. While this pattern might occur simply because the response bias results from the probability manipulation directly, it is also consistent with a process in which items appearing at cued locations are given preferential access to decision-making processes. This process leads to a performance advantage while observers are not pressured to respond too quickly. As observers come under more pressure to respond quickly, the preferential access they had afforded items appearing at cued locations becomes a bias to respond quickly, and not necessarily accurately, to potentially unprocessed targets appearing at the cued location.

## ACKNOWLEDGMENTS

This research was funded by a grant from the Natural Sciences and Engineering Research Council of Canada awarded to P. McCormick. Part of this research was generated in an unpublished Honours thesis by L. Francis presented to St. Francis Xavier University. We would like to thank Chip Folk and Hermann J. Müller for valuable comments on an earlier version of this manuscript and Melissa Pye for technical assistance.

## APPENDIX A
**Mean Response Times (ms) and Error Rates (in Brackets) for Normal Instructions and Deadline Intervals for Valid and Invalid Cues from Experiment 1 (Exogenous Orienting)**

| | Cue Condition | | |
|---|---|---|---|
| Cut-off | Valid | Invalid | Difference |
| Normal Instructions | 698 (.024) | 742 (.018) | 44 (-.006) |
| 590 ms | 440 (.030) | 458 (.029) | 18 (-.001) |
| 560 ms | 415 (.040) | 425 (.055) | 10 (.015) |
| 530 ms | 404 (.048) | 413 (.063) | 9 (.015) |
| 500 ms | 392 (.065) | 406 (.064) | 14 (-.001) |
| 470 ms | 381 (.073) | 393 (.075) | 12 (.002) |
| 440 ms | 369 (.088) | 376 (.106) | 7 (.018) |
| 410 ms | 348 (.149) | 360 (.154) | 12 (.006) |
| 380 ms | 336 (.203) | 345 (.205) | 9 (.002) |
| 350 ms | 308 (.240) | 308 (.263) | 0 (.023) |
| 320 ms | 274 (.358) | 279 (.348) | 5 (-.010) |
| 290 ms | 249 (.381) | 243 (.388) | -6 (.007) |
| 260 ms | 222 (.406) | 227 (.419) | 5 (.013) |
| 230 ms | 199 (.443) | 195 (.416) | -5 (-.027) |
| 200 ms | 175 (.450) | 168 (.475) | -7 (.025) |

## APPENDIX B
**Mean Response Times (ms) and Error Rates (in Brackets) for Normal Instructions and Deadline Intervals for Valid and Invalid Cues from Experiment 2 (Endogenous Orienting)**

| | Cue Condition | | |
|---|---|---|---|
| Cut-off | Valid | Invalid | Difference |
| Normal Instructions | 447 (.105) | 464 (.109) | 17 (.004) |
| 590 ms | 416 (.104) | 443 (.131) | 27 (.027) |
| 560 ms | 406 (.096) | 422 (.156) | 16 (.060) |
| 530 ms | 396 (.123) | 414 (.148) | 18 (.025) |
| 500 ms | 397 (.128) | 400 (.144) | 3 (.016) |
| 470 ms | 391 (.155) | 396 (.154) | 5 (-.001) |
| 440 ms | 371 (.164) | 382 (.216) | 11 (.052) |
| 410 ms | 355 (.226) | 372 (.189) | 17 (-.037) |
| 380 ms | 342 (.259) | 360 (.275) | 18 (.016) |
| 350 ms | 314 (.314) | 324 (.308) | 10 (-.060) |
| 320 ms | 279 (.369) | 302 (.369) | 23 (.000) |
| 290 ms | 254 (.425) | 291 (.429) | 37 (.004) |
| 260 ms | 233 (.456) | 248 (.389) | 15 (-.067) |
| 230 ms | 212 (.495) | 246 (.459) | 34 (-.036) |
| 200 ms | 189 (.473) | 216 (.420) | 27 (-.053) |
